# Rapid Raman Spectroscopic Analysis of Stress Induced Degradation of the Pharmaceutical Drug Tetracycline

**DOI:** 10.3390/molecules25081866

**Published:** 2020-04-17

**Authors:** Christian Domes, Timea Frosch, Juergen Popp, Torsten Frosch

**Affiliations:** 1 Leibniz Institute of Photonic Technology, 07745 Jena, Germany; christian.domes@leibniz-ipht.de (C.D.); timea.frosch@uni-jena.de (T.F.); juergen.popp@ipht-jena.de (J.P.); 2 Institute of Physical Chemistry, Friedrich Schiller University, 07743 Jena, Germany; 3 Abbe Center of Photonics, Friedrich Schiller University, 07745 Jena, Germany

**Keywords:** resonance Raman spectroscopy, drug monitoring, active pharmaceutical ingredients, antibiotics, drug degradation, decomposition of pharmaceutical products, storage stress test

## Abstract

Stress factors caused by inadequate storage can induce the unwanted degradation of active compounds in pharmaceutical formulations. Resonance Raman spectroscopy is presented as an analytical tool for rapid monitoring of small concentration changes of tetracycline and the metabolite 4˗epianhydrotetracycline. These degradation processes were experimentally induced by changes in temperature, humidity, and irradiation with visible light over a time period of up to 23 days. The excitation wavelength *λ*_exc_ = 413 nm was proven to provide short acquisition times for the simultaneous Raman spectroscopic detection of the degradation of tetracycline and production of its impurity in small sample volumes. Small concentration changes could be detected (down to 1.4% for tetracycline and 0.3% for 4-epianhydrotetracycline), which shows the potential of resonance Raman spectroscopy for analyzing the decomposition of pharmaceutical products.

## 1. Introduction

Antibiotics are used for the treatment of a broad range of infections caused by gram-positive and gram-negative bacteria [1]. The group of tetracyclines was discovered in 1947 as a product of the *Streptomyces* genus of *Actinobacteria* [2]. With their large number of derivatives, such as tetracycline (TC), chlortetracycline, and oxytetracycline, these broad-spectrum antibiotics are used in human medicine [3,4] and in veterinary medicine [5,6]. The derivatives doxycycline, minocycline, and vibramycin are widely used in human medicine, where the indication field of doxycycline ranges from severe infections with *Bacillum anthracis* to malaria prophylaxis [7]. Tetracyclines are available in various pharmaceutical formulations: tablet, powder for suspension, capsule or syrup [8]. Due to inadequate long-term storage, dehydration and isomerization can take place, as already investigated [9,10,11,12]. The degradation products 4-epitetracycline (ETC), anhydrotetracycline (ATC), and 4-epianhydrotetracycline (EATC) change the effectiveness of the active pharmaceutical ingredient (API) due to their pharmaceutical inactivity and are partly toxic [13,14,15]. In the pharmaceutical industry, impurities have to be controlled in the pharmaceutical end product, where multiple parameters, such as pH value, oxidation, temperature, humidity, and photo-stability have to be considered and investigated [16,17,18]. Thus, there is an urgent need for monitoring the specific metabolite composition quickly and with high chemical selectivity. Especially in poorly developed countries, the quality of the pharmaceuticals is often not ensured due to frequent counterfeit or substandard products on the one hand [19,20,21], and improper storage circumstances on the other hand. A study from Nigeria [22] showed that only one capsule from seven analyzed samples did not contain any degradation products of tetracycline.

The established techniques for the separation and quantification of drugs and their degradation products, as well as their pharmacokinetics, are mainly chromatography-based (e.g., ultra-performance liquid chromatography (UPLC™) [23,24], liquid chromatography–mass spectrometry [LC-MS(/MS)] [25,26], and liquid chromatography nuclear magnetic resonance (LC-NMR) [27]). These techniques take a long time from the test to the result, are expensive, strictly lab-based, need consumables, and require trained personal. Thus, there is a need for new methods, which can overcome some of these drawbacks while maintaining the sensitivity and selectivity for the analyte determination.

In recent years, Raman spectroscopy has been developed for the analysis of pharmaceutical samples [28,29,30,31,32,33,34,35,36,37,38,39]. This technique [40] provides several advantages as a non-invasive [41], label-free [42,43,44,45,46,47,48], fast and sensitive [49,50,51,52,53] method that can be applied on-site [54,55,56,57,58,59,60] and for the simultaneous analysis of various metabolites. These abilities show further potential for in-line and real-time quality assessment in pharmaceutical production processes. This work investigates the stability of the API during stress treatments which are typical for inadequate storage, by monitoring the degradation of TC and its metabolite EATC in a pharmaceutical formulation.

## 2. Materials and Methods

### 2.1. Sample Preparation

**Figure 1:**
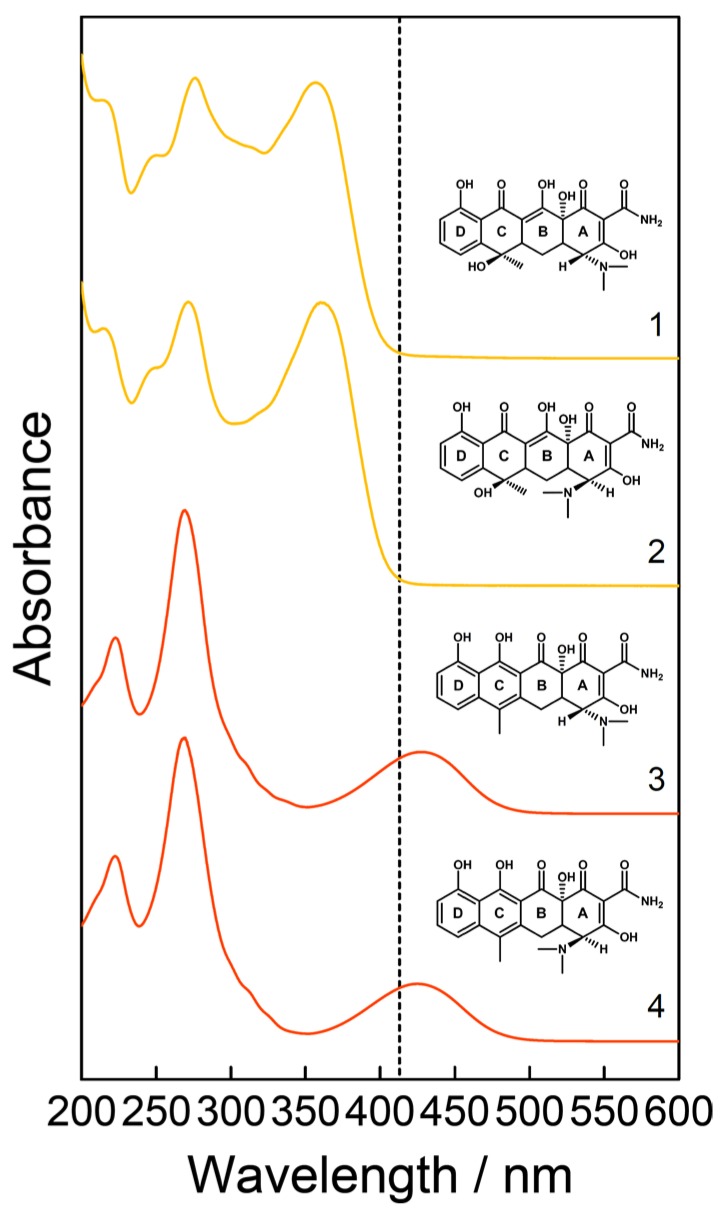
Schematic chemical structures and absorption spectra of a 0.1 mM aqueous solution of tetracycline (TC, 1) and its impurities 4-epitetracycline (ETC, 2), anhydrotetracycline (ATC, 3), and 4-epianhydrotetracycline (EATC, 4). The applied laser excitation wavelength *λ*_exc_ = 413 nm is depicted as vertical short-dashed line. This wavelength can be tuned into the electronic absorption band of the chromophores of (E)ATC, where strong resonance Raman enhancements can be achieved for the vibrational modes that are coupled to these electronic transitions. This resonance effect can be exploited for the sensitive detection of these impurities.

TC (tetracycline hydrochloride, 98%) and its metabolites ETC (4-epitetracycline hydrochloride, 97.2%), ATC (anhydrotetracycline hydrochloride, 98%), and EATC (4-epianhydrotetracycline hydrochloride, 96%) were purchased from Sigma–Aldrich (Merck KGaA, Darmstadt, Germany) and used without further purification (Figure 1). Capsules containing the API tetracycline were ordered from Dr. August Wolff (Dr. August Wolff GmbH & Co. KG, Bielefeld, Germany). The hard capsules were opened and the powder (mass = 250 mg) was used for further experiments.

Two different treatments were applied to the pharmaceutical samples, where the first one was thermal and humidity stress. Here, the samples were stored at 70 °C and relative humidity of 70% for up to 23 days in a climate chamber [HPP110 (Memmert, Schwabach, Germany)] for stress treatment. To simulate an inadequate long-time storage with accelerated stability test, the Arrhenius kinetics with equation (1) can be applied [61], where k is the rate factor, A the pre-exponential factor, $E_{A}$ the activation energy, and RT the product of temperature and the universal gas constant.

(1) $$k=A\cdot \exp\left(-\frac{E_{A}}{RT}\right)$$

In general, and in this study, an activation energy of 15 kcal/mol was assumed [17]. Thus, a treatment for approximately 21 months at 25 °C can be simulated with the exposure of 70 °C for 23 days.

The aim of the second experiment was to test the photo-stability of the samples. In accordance with ICH photo-stability guidelines [62], the pharmaceuticals were exposed to visible light (400–760 nm) over a time period ranging from 4 to 37 h, using a LED lamp [SANlight M30, (SANlight GmbH, Bludenz, Austria)] as a light source. The recommended exposure was an overall illumination of more than two (here up to ten) times 1.2 Mlux⋅h.

For both experiments, the first sample was kept in the dark without any treatment as reference for further investigations. After the treatment, the powder was weighted and a 100 mM stock solution (calculated for TC) was prepared using ultra-clear water from a feed system [SG Water GmbH, (Siemens, Barsbuettel, Germany) with κ > 0.06 µS/cm]. For quantification, a dilution series of 0–2 mM EATC and 10–8 mM TC (always a mixture with overall concentration of 10 mM) was used, which grants the prevailing condition during the prediction of a 10 mM solution of the pharmaceutical solutions.

### 2.2. Optical Spectroscopy

Resonance Raman spectra were acquired with a Raman microscope [LabRAM, Jobin Yvon HR800 (Bensheim, Germany)], using a 10× objective and 413 nm as the excitation wavelength (laser power at the samples: 33 mW). A 300 L/mm grating and an exposure time of 60 s (two accumulations) was used to measure three replicates of each sample solution. To avoid any photo-degradation during the measurements, the samples were constantly stirred in a rotational cuvette (V = 400 µL). The solid samples of the investigated substances were also measured, using a 100× objective, a 600 L/mm grating, and an exposure time of 30 s with two accumulations. With help of an OD2 filter, the resulting laser power at the sample was only 120 µW, to avoid any photo-degradation and heating events.

The absorption and Fourier transform (FT-) Raman spectra were measured with a Cary 5000 system (Varian, Darmstadt, Germany) and a Ram II spectrometer (Bruker, Bremen, Germany) with an Nd:YAG laser with excitation wavelength of 1064 nm, respectively.

### 2.3. Data Preprocessing and Quantification

All preprocessing and analysis of the raw Raman data was performed in statistical programming GnuR 3.6.1 [63]. The packages ′signal′ [64], ′EMSC′ [65], ′baseline′, [66], and ′minpack.lm′ [67] were utilized and their functions were complemented. First, a wavenumber-calibration [68] was performed with the Raman signals from acetonitrile and toluene. Then, the Raman data were truncated to the wavenumber region of interest (1150-1750 cm^−1^) and a Savitzky-Golay smoothing (*p* = 2, *n* = 5) was applied. Afterwards, the resulting spectra were scatter corrected with the extended multiplicative signal correction (EMSC), using the mean spectra of each sample as a reference (degree = 6), and baseline corrected, using a third-order polynomial fit. For quantification, the spectra were normalized to the Raman band of water at approximately 1655 cm^−1^. The difference spectra were calculated using the mean water spectrum as a reference and a Gaussian peak profile was fitted to the signals of TC and EATC at approximately 1455 and 1515 cm^−1^, respectively. The resulting peak areas were correlated with the corresponding concentrations and showed a linear relation. Thus, the peak areas were used for the prediction of the drug composition.

### 2.4. Density Functional Theory Calculation for Vibrational Assignment of Raman Marker Bands

For a better understanding of the assignment and an interpretation of the Raman marker bands used for the quantification, the molecular structures were optimized and vibrational modes and Raman activities were calculated with density functional theory (DFT) using Gaussian 09 [69]. The hybrid exchange correlation functional with Becke’s three-parameter exchange functional (B3) [70] slightly modified by Stephens et al. [71] coupled with the correlation part of the functional from Lee, Yang, and Parr (B3LYP) [72] and Dunning’s triple correlation consistent basis sets of contracted Gaussian functions with polarized and diffuse functions (cc-pVTZ) [73] were applied. For alignment, the wavenumber positions of the FT-Raman peaks (threshold: 20% of the maximum intensity) were scaled to the scattering activities of the calculation. The frequency-scaling factor was calculated by minimizing the mean average error (MAE), and an intensity correction [74] was estimated. Finally, the scaled scattering activities were fitted with a Lorentzian Peak profile and a full width at half maximum (FWHM) of 27 cm^−1^ to simulate Raman bands with finite resolution.

## 3. Results and Discussion

The established, aforementioned methods in pharmaceutical quality assurance require large sample volumes, are time-consuming, labor-intensive and destructive [75]. Thus, there is a need for new sensitive and non-destructive techniques. Tetracycline and its derivatives were investigated with Raman techniques [76,77,78,79], but no detailed studies were performed yet, which explicitly deal with the quantification of its impurities. In this study, the potential of resonance Raman spectroscopy is demonstrated as an alternative method for the sensitive detection of small concentration changes during the stress treatment of TC.

### Resonance Raman Spectroscopy for Quantification of Concentration Changes

**Figure 2:**
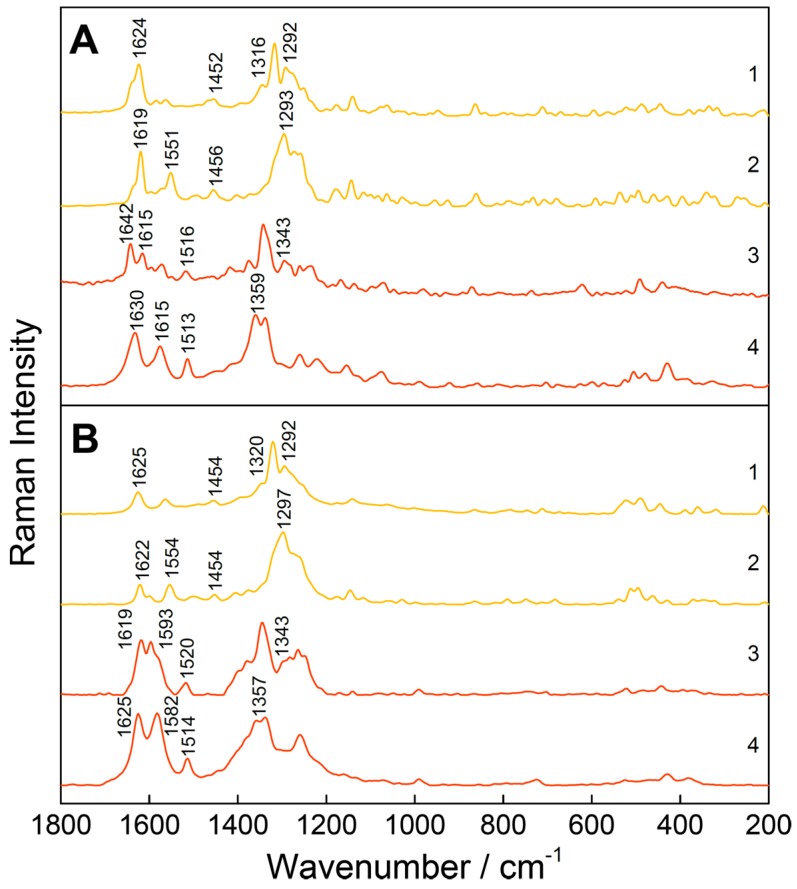
FT-Raman (**A**) and resonance Raman spectra (**B**, *λ*_exc_ = 413 nm) of TC (1), ETC (2), ATC (3) and EATC (4) in solid state. By using *λ*_exc_ = 413 nm as the excitation wavelength, a relative decrease of the Raman signals of TC and ETC and a relative enhancement of the Raman bands of ATC (and EATC) was observed in the range 1550–1650 cm^−1^ with respect to the highest peak, respectively. For all samples, the strongest Raman marker bands are displayed and detailed vibrational studies for TCs can be found in the literature [77,80,81]. Here, due to the different wavenumber resolution [0.96 (**A**) vs. 2.85 cm^−1^ (**B**)] the peak position can differ slightly.

**Figure 3:**
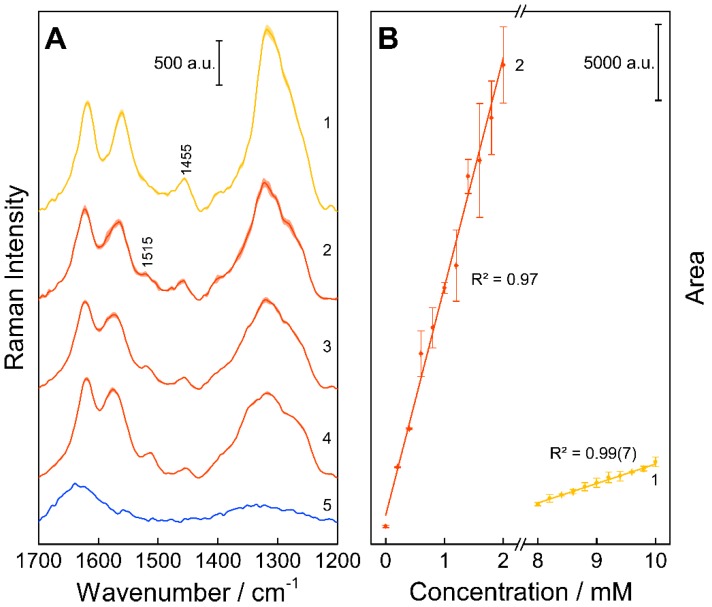
Representative resonance Raman spectra (**A**) of 10 mM TC (1) and 2 mM* EATC (2) used for predicting the contribution of the 10 mM solutions of the stress-treated drugs. Here, the severest conditions for temperature/humidity (23 days, 70% relative humidity and 70 °C (3)) and light treatment (12 Mlux⋅h (4)) are shown. Additionally, the water reference spectrum (5) can be found, which was used for the calculation of the difference spectra. The utilized signals for the quantification of the substances in the Raman spectra acquired with *λ*_exc_ = 413 nm are marked. Small changes can be observed in the Raman peaks due to the treatments. The average Raman spectra and their deviation are depicted. Regression lines (**B**) with corresponding coefficients of determination for the quantification of TC (1) and EATC (2). The calibration points were defined with the peak area of the respective fitted Raman marker bands at 1455 and 1515 cm^−1^ for TC and EATC using *λ*_exc_ = 413 nm as the excitation wavelength and result in a good linear relation. *As mentioned earlier, a mixture of TC and EATC was used for quantification (10 + 0 mM up to 8 + 2 mM). Therefore, the Raman signal of TC is also present in (2).

First, an optimal excitation wavelength was chosen for the simultaneous detection of TC and its impurities ETC, ATC, and EATC. With an absorption band at around 430 nm for ATC and EATC (Figure 1), the excitation wavelength *λ*_exc_ = 413 nm results in a strong enhancement of the Raman signals of the investigated impurities in the wavenumber range 1550–1650 cm^−1^ (Figure 2). In this study, the concentration changes of TC and EATC were monitored. Regarding the FT- or resonance Raman spectra of the solid state of these analytes (Figure 2) the wavenumber region between 1200 and 1400 cm^−1^ seems to be promising to tackle this issue. The actual pharmaceutical samples were measured in water and the Raman bands suffer from broadening (Figure 3). Hence, the well separated Raman signals at 1455 and 1515 cm^−1^ (Figure 3) were monitored during the degradation studies. The Raman peaks were fitted with Gaussian peak profiles and the respective peak areas were used for the quantification.

**Figure 4:**
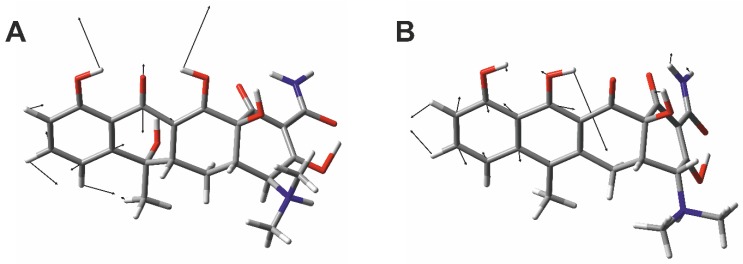
Assignment of the vibrational modes of the Raman marker bands. Atomic displacements of the Raman bands of TC at 1455 cm^−1^ (**A**) and EATC at 1515 cm^−1^ (**B**) is shown, respectively. The color code for the individual atoms is: hydrogen (white), carbon (grey), oxygen (red), and nitrogen (blue).

For a better understanding of these marker bands, DFT calculations were aligned with the experimental FT-Raman spectra (Figure 2A and Figure S1) and their vibrational modes (Figure 4) were analyzed. The vibration at 1455 cm^−1^ can be construed as a combination of a symmetric C=O-stretching of the *C*-ring system, an OH-wagging of the hydroxyl group of the *D*- and *B*-ring system, and a CC-stretching and CH-rocking vibration located at the *D*-ring system (Figure 1, Figure 4A). The vibration at 1515 cm^−1^ can be considered as an OH-wagging of the hydroxyl group of the *C*-ring system combined with a CC-stretching and CH-rocking vibration located at the *D*- and *C*-ring system (Figure 4B) for TC and EATC, respectively.

**Figure 5:**
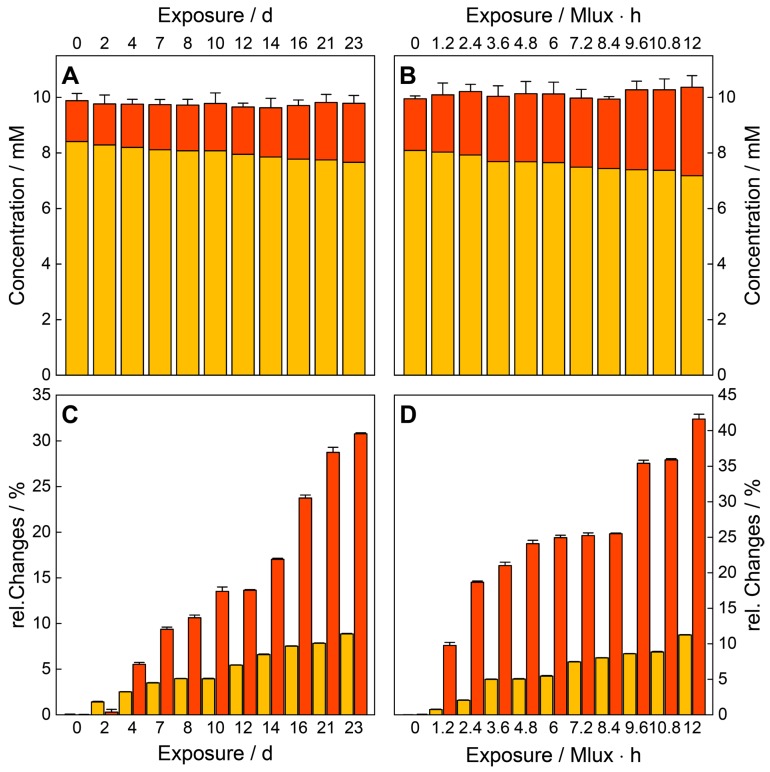
Contribution of the investigated tablet for TC (orange) and EATC (red), under treatment with humidity/temperature (**A**) and light (**B**). The sum of both mean values (with its respective error bars) deviates only slightly from the total concentration of 10 mM. Due to the exposure, a decrease (increase) of the concentration of TC (EATC) is observed with time. The general trend of the concentration changes is independent of the treatment, but the values of relative production of EATC (relative to the first concentration) are up to five times higher than the ones for the degradation of TC. The two treatments with humidity and temperature (RH/RT, **C**) and light (Vis, **D**) are shown. Light exposure results in 1.5 times stronger decrease (increase) of TC (EATC), compared to the treatment with temperature/humidity, respectively.

The fitted peak areas of the concentration series were correlated with their concentration values and the resulting calibration curve was used for predicting the sample concentrations (Figure 3B). With increasing storage time and light exposure, a linearly decreasing concentration (degradation) of TC and increasing concentration (formation) of EATC could be detected (Figure 5A,B). Since only small changes occurred during the stress treatment (Figure 4), the relative values were calculated, using equation (2) with $\Delta c=c_{0}-c_{i}$ as the absolute changes during the treatment and $c_{0}$ and $c_{i}$ as the concentrations of the reference and the respective sample.

(2) $$c_{d}=\frac{\left|\Delta c\right|}{c_{0}}\;\mathrm{and}\;c_{p}=\frac{\left|\Delta c\right|}{c_{i}}$$

The relative concentration values of EATC increase for the temperature and humidity treatment linearly up to 30% after 23 days, while the concentration values of TC decrease relatively by 9% during the treatment. Thus, the epimerization and dehydration reactions were performed and accelerated due to the stress exposure.

The results of the photo-stability test follow the same trend, with up to 1.5 times higher values for all samples (see Figure 5C,D). After treatment with 12 Mlux⋅h, 42% EATC were produced and 14% TC degraded, respectively. Thus, it could be shown in the literature [82,83] that the exposure to light in a relatively humid environment can cause the formation of singlet oxygen, which could, in interaction with TC, generate hydrogen peroxide, a photo-toxin. In combination with the higher temperature provided by the illumination lamp, this treatment causes stronger degradation changes in the drug composition.

## 4. Conclusions

This work illustrates the potential of resonance Raman spectroscopy for the monitoring of small concentration changes of TC and its impurity EATC during two different stress treatments. Strong resonance Raman signals could be acquired by use of the excitation wavelength *λ*_exc_ = 413 nm and a linear relationship between the concentration values and the peak areas were achieved for stable quantification. The direct Raman spectroscopic measurement features short measurement times and minimal effort for the quantification of TC and EATC, in comparison to standard techniques.

The detection of only minor concentration changes of API and its inactive metabolites during the performed experiments demonstrate the high potential of resonance Raman spectroscopy as a rapid analytical tool and provides the foundation for further degradation studies and pharmaceutical applications.

## Supplementary Materials

The following are available online at https://www.mdpi.com/1420-3049/25/8/1866/s1, Figure S1. Alignment of the experimental FT-Raman spectra of TC.
