# Supplementary material for: Rapid Raman Spectroscopic Analysis of Stress Induced Degradation of the Pharmaceutical Drug Tetracycline
Source: Molecules. 2020 Apr 17;25(8):1866. doi: 10.3390/molecules25081866 (PMC7221697; doi:10.3390/molecules25081866)
Supplement: Supplementary file 1 [file molecules-25-01866-s001.pdf]

Supplementary Information

Electronic Supplementary Information 1

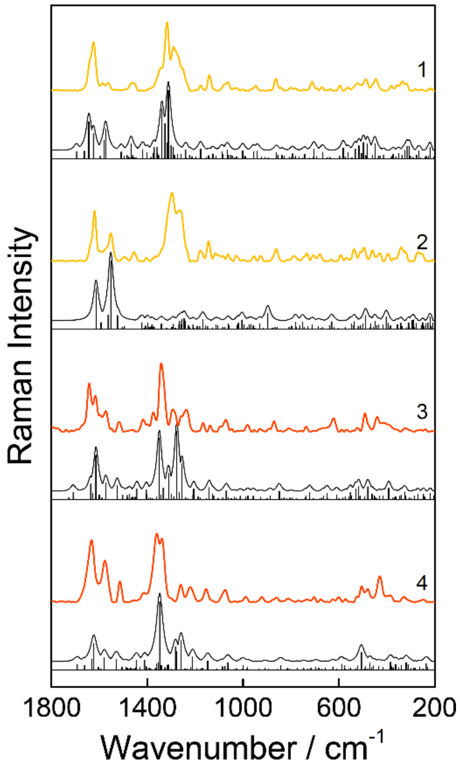

Figure S1. Alignment of the experimental FT-Raman spectra of TC (**1**), ETC (**2**), ATC (**3**), and EATC (**4**) with the calculated scattering activities (calculation method: B3LYP/cc-pVTZ) and their Lorenzian peak profile (FWHM = 27 cm^{-1}). Here, the scaling factors and the resulting MAE values for the investigated wavenumber region (1800–200 cm^{-1}) are 0.98 and 1.23 (**1**), 0.93 and 1.92 (**2**), 0.98 and 2.47 (**3**), and 0.98 and 2.50 (**4**).
